# Serum albumin level difference in burn injury after tangential excision: A prospective cohort study

**DOI:** 10.1016/j.amsu.2020.02.007

**Published:** 2020-02-26

**Authors:** Hardisiswo Soedjana, Setiagung A. Bowo, Nandita Melati Putri, Theresia Risa Davita

**Affiliations:** a Head of Division of Plastic Reconstructive and Aesthetic Surgery Department of Surgery, Dr. Hasan Sadikin General Hospital, Padjadjaran University, West Java, Indonesia; b Division of Plastic Reconstructive and Aesthetic Surgery, Department of Surgery, University of Indonesia, Jakarta, Indonesia; c Division of Plastic Reconstructive and Aesthetic Surgery Department of Surgery, Dr. Hasan Sadikin General Hospital, Padjadaran University, West Java, Indonesia

**Keywords:** Albumin, Burn injury, Tangential excision

## Abstract

**Background:**

Tangential excision in burn patients results in blood loss, with an average of 100 ml per 1% total body surface area (TBSA) excised. This substantial blood loss will be accompanied by decreasing serum albumin concentration, increasing inflammation, capillary leakage, and surgical stress. The importance of maintaining albumin level in burn injury is essential for wound healing, decreasing the susceptibility of sepsis, and preventing acute respiratory distress syndrome, the leading causes of death in burn injuries.

**Methods:**

This was a prospective cohort study at our plastic surgery center in Bandung, West Java, Indonesia from January 2018. Serum albumin were sampled taken preoperatively and postoperatively after 24 h. Correlation to the percentage of burn tissue excised was analyzed.

**Result:**

Twenty-eight patients with burn injury were enrolled as study subjects. There was a significant drop in serum albumin after tangential excision surgery compared to prior surgery in burn patients with mean reduction of 8.6 ± 7.3% (P-value < 0.05) to the mean postoperative albumin value is 0.15 ± 0.1 g/dL. The albumin drop was correlated with blood loss (r = 0.326, P-value < 0.05) but not with the excision area (r = 0.196), length of surgery (r = −0.077) and TBSA (r = −0.213). Strong correlation was shown between excision area with the amount of blood loss (r = 0.567, P-value = 0.001).

**Conclusion:**

Tangential excision leads to a reduction in postoperative serum albumin concentration. The magnitude of albumin reduction strongly correlated with the amount of blood loss amount during the procedure.

## Highlights

• Burn injury causes hypoalbuminemia.
• Tangential excision is a surgical routine in burn injury as sepsis prevention.
• Tangential excision leads to further serum albumin reduction postoperatively.
• Albumin level changes affected by various mechanism and variables in surgery.
• Serum albumin level reduction increases various postoperative complication risks.

## 1 Introduction

Tangential excision is a debridement method to prevent sepsis in burn injuries. Most burn centers have limitations in each operative session to excise up to 10–20% of Total Body Surface Area (TBSA) although some may perform larger debridement (>20% of TBSA) in a single procedure. Clinical studies showed that the average bleeding in adult tangential excision was 100 mL for every 1% TBSA [1].

Plasma protein contains 55–60% albumin as the major protein carrier. Albumin has the main function of maintaining normal plasma colloid oncotic pressure. In normal physiological conditions, about two-thirds of the total body albumin pool is in the extravascular space. Albumin is produced exclusively in the liver, representing 50% of its protein synthesis, and can be upregulated to threefold if the oncotic pressure decreases [2,3]. Albumin degradation occurs in various organs about 5% per day with a half-life of 19 days [3].

The decrease of albumin occurs rapidly after surgery, it will drop to its minimum concentration in 24 h postoperative, and then reach its stable state in the next 48 h [4]. Early postoperative albumin depletion occurs due to the combination of surgical stress and blood loss, also from altered metabolism or dilution. Fluid redistribution into the third space due to capillary leakage related to systemic inflammatory response becomes the most important factor to contribute to the early decrease of albumin level. The depletion of serum albumin concentration ≥1 g/dL in 24 h postoperative is associated with a threefold increased risk of overall postoperative complications [5]. This happens not only in burn injury but also in colorectal surgery with every 5 × 10^−4^ g/dl decrease in albumin concentration is progressively associated with an increased risk of complications [6].

One of the most important albumin functions is to maintain oncotic pressure. Reduction of serum albumin level can predispose patients to decreased immune response, wound healing disruption, malnutrition, drug transport alteration, and increased risk of infection [7,8]. Severe hypoalbuminemia (≤2 g/dl), was strongly associated with greater burn severity and 33% higher in mortality rate. A cut-off point of 1.95 g/dl serum albumin concentration was a strong predictor of poor prognosis [8]. Serum albumin less than 3 g/dl was associated with more severe and frequent postoperative complications in major surgeries [9,10].

Acute respiratory distress syndrome (ARDS) is the most common cause of death (53.5%), followed by sepsis (42.3%) at our hospital from 2012 to 2015. Some of the predisposing factors for ARDS development are decreased oncotic pressure from hypoalbuminemia and increased vascular permeability due to inflammation which can lead to extravasation of intravascular fluid into the interstitial space [11].

This study aims to investigate the change of serum albumin concentration in tangential excision pre and postoperatively and its correlation to the extent of the excision area. We believe this study would be the fundamental data on the importance of perioperative monitoring albumin level in tangential excision procedures and may contribute to the development of better strategies to prevent burn injury complications.

## 2 Methods

This study was a prospective cohort study at our plastic surgery center in Bandung, West Java, Indonesia from January 2018 to June 2018. All family/patients were informed about the research project during their first encounter at the surgery department. Subjects are included after signing a written informed consent. This research is in line with the STROCSS criteria [12]. The Unique Identifying Number (UIN) of this study is researchregistry5095 in Research Registry registration, in accordance with the declaration of Helsinki.

### 2.1 Inclusion criteria

- Adult patients (17–45 years old) with extent >20% burn injury in grade 2B and 3.
- Patients who come within 24 h after burn injury
- Good nutrition status with normal Body Mass Index (BMI 18–25)
- Minimum preoperative hemoglobin concentration of 10 gr%
- Excised area 5–20% TBSA within the third to the fifth day after burn injury

### 2.2 Exclusion criteria

- Patients who received an intraoperative transfusion of plasma or albumin serum,
- Patients who received intraoperative skin grafts
- Pregnant patients

Tangential excision procedure was done by the first author who has more than 20 years of experience as a plastic surgeon. Surgery was assisted with other authors as well. Serum albumin was sampled in fasting state, within 0–6 h before surgery and 24 h after surgery to avoid bias. The sample was measured in our laboratory center. Surgery duration was documented by the anesthesiologist. Intraoperative blood loss was estimated as a joint decision from the anesthesiologist and the main surgeon by measuring the bleeding tube and counting the soaked gauze materials. Some demographic data such as age, TBSA, gender, and causes of trauma were noted.

Data were analyzed using SPSS ver. 20.0. Serum albumin concentration were analyzed using paired *t*-test. The normality test was done using the Shapiro-Wilk test. Data correlation was obtained using Pearson's test. A P-value of <0.05 was regarded as statistically significant.

## 3 Results

Twenty-eight patients with burn injury were enrolled as study subjects comprised of 20 male patients (71.43%) and 8 female patients (28.57%) with the characteristic shown in Table 1. The sources of trauma are from a direct flame in 20 patients (71.4%), electrical in 5 patients (17.9%), and scald in 3 patients (10.7%) (see Table 2).

**Table 1:** Characteristic of the patients.

Variable	Mean	Range
Age (years)	33.9 ± 10.3	18–45
BMI	22.0 ± 1.9	18,4-25
TBSA (%)	29.6 ± 12.8	20–70
Excision (%)	9.6 ± 4.9	5–20
Blood loss (cc)	311.8 ± 154.9	100–650
Length of surgery (minute)	79.1 ± 25.8	45–120
Albumin preoperative (g/dl)	1.7 ± 0.3	1.1–2.6
Albumin postoperative (g/dl)	1.5 ± 0.3	0.9–2.6
Percentage of depletion albumin serum (%)	8.6 ± 7.3	0–31.3

**Table 2:** The extent of excised area.

Extent of excised area [10]	n (%)
5%–10%	20 (71.4)
11%–15%	4 (14.3)
16%–20%	4 (14.3)

Table 1 showed the mean of variable collected data. There was a significant difference from the reduction of serum albumin level pre and post tangential excision surgery in burn patients (P-value < 0.05) with mean delta albumin of 0.15 ± 0.1 g/dL.

Sex, source of trauma, and extent of excision area had no significant association with the reduction of serum albumin level in tangential excision (P-value > 0.05). Although the extent of excision area in tangential excision procedure correlated to blood loss (P-value < 0.05), there was no significant correlation found between the extent of the excised area with the reduction of serum albumin concentration. However, the depletion of serum albumin was correlated to intraoperative blood loss (P-value < 0.05) as shown in Table 3.

**Table 3:** Correlation of percentage serum albumin drop.

Variable	Percentage of serum albumin drop	
	R	P-value
Excised area	0.196	0.159
**Blood loss**	**0.326**	**0.045**
TBSA	−0.213	0.139
Length of surgery	−0.77	0.345

Almost all of the patients who underwent tangential excision had lower serum albumin level after surgery compared to prior surgery. The mean percentage depletion of albumin in this study had a positive correlation with intraoperative blood loss (P-value < 0.05).

## 4 Discussion

Serum albumin reduction is highly associated with the increased mortality risk in major burn injuries [13]. Studies showed that in large burn injury (20–30% TBSA), mediators such as prostaglandins, histamine, leukotrienes, bradykinin, vasoactive amines, platelet activation products, and complement are produced largely at the burn site [1,[14], [15], [16]]. The inflammatory mediators induce vascular permeability that plays a role in protein loss into the interstitial space, which results in intravascular fluid, electrolytes and protein depletion [[14], [15], [16]]. Inflammation in burn patients itself decreases albumin synthesis because the liver needs to produce acute-phase proteins [17]. The depletion of serum albumin is used for well-established nutrition assessment and predicting complications [9]. Alberti et al. recently found that major digestive surgery decreased serum albumin postoperatively which was shown due to systemic and tissue inflammation, alteration in vascular permeability and dilution of intravenous infusion [10].

Other studies showed that hypoalbuminemia has a strong association to the extent of affected TBSA; the wider the TBSA of burn injury, the higher the mortality rate [16,17]. The mean extent of burn injury in this study was 29.6 ± 12.8% TBSA. The preoperative serum albumin mean was 1.7 ± 0.3 g/dl. This indicates that hypoalbuminemia was already present in extensive burn injury victims prior to surgery. Ishida et al. concluded that hypoalbuminemia in burns patients can occur as a result of high fluid resuscitation and the increased vascular permeability in burn injury rather than due to lack of nutritional supply. Thus, serum albumin level can be used as a marker for trauma severity, rather than just as a nutritional status [17]. Inflammation in burn patients is caused not only by the burn injury itself, but also can be induced by infection, various surgery procedures, and underlying diseases [16,17].

It has been described from the previous studies that bleeding in tangential excision surgery can be predicted from the extent of the excision area. In adults, every 1% of TBSA excised results in an average of 100 mL blood loss. However, blood loss can be reduced by almost 50% from the prediction by using adrenalin diluted soaked gauze and pressure after the dead skin has been excised. Full-thickness excision of devitalized tissue to the level of muscle fascia with electrocautery also helps to limit blood loss [1,18]. The mean area of excision in tangential excision surgery in this study was 9.6 ± 4.9% TBSA and intraoperative blood loss mean was only 311.8 ± 154.9 cc from adapting the techniques. The procedure was done during the third to fifth day after burn injury. There was a correlation between the extent of the excised area to blood loss (P-value < 0.05).

The local and systemic effect of mediators released from burned tissue is reduced by tangential excision of the devitalized tissue, therefore reducing the progression of pathophysiologic derangements. Tangential excision has been shown as a promising procedure to reduce sepsis risk, length of hospital stays, and even mortality rate [16,18,19]. However, there was a distinctive reduction of albumin serum after this procedure. Mean postoperative serum albumin was 1.5 ± 0.3 g/dl. This reduction showed a significant difference compared to the serum albumin level before surgery (P-value < 0.05). The decreased of serum albumin level represents an immediate response to surgical stress, which occurs rapidly after the surgery. It will drop to its minimum concentration on the first day, then it will become stable on the third day after surgery. This depletion associated with the increase of other surgical stress biomarkers like procalcitonin, lactate and c-reactive protein [4]. The mechanisms of early postoperative albumin depletion are due to the combination of altered metabolism, surgical stress, blood loss, fluid dilution, and most importantly capillary leakage that causing fluid redistribution into the third space. Serum albumin level usually decreases more than 75% in the early postoperative phase and appears to be related to the systemic inflammatory response [5]. This rapid drop in serum albumin may worsen the prognosis of burn patients.

There was no significant correlation between the extent of the excised area to albumin reduction. Blood loss was correlated to serum albumin level reduction (P-value < 0.05). Albumin depletion occurs not only from albumin loss along with the bleeding but also from intraoperative hemodilution. This decreased in albumin concentration is not easily corrected as the administration of additional human plasma albumin through intravascular infusion will be redistributed into the extravascular compartment within 24 h [17].

## 5 Conclusion

Tangential excision causes a reduction of serum albumin level postoperatively. The albumin depletion has a positive correlation to the amount of blood loss and the extent of the excision area, though only the amount of blood loss that significantly correlated. Further studies are needed to observe other perioperative factors that may influence the reduction of serum albumin in burn patients. These data are expected to be the foundation for further work and be a useful addition to the scientific literature to provide better medical care to burn patients.

## Provenance and peer review

Not commissioned externally peer reviewed.

## Ethical approval

The Health Research Ethics Committee of the central public hospital, Dr. Hasan Sadikin General Hospital Bandung, after the discussion and evaluation on February 27th, 2018, hereby decided and agreed on this research protocol. The research was registered in Research Registry on August 30th, 2019 using UIN researchregistry5095.

## Sources of funding

None.

## Author contribution

Hardisiswo Soedjana: concepts, design, surgeon, definition of intellectual content, literature research, clinical studies, experimental studies, data acquisition, data analysis, manuscript editing & review, guarantor.

Setiagung A Bowo: definition of intellectual content, literature research, clinical studies, experimental studies, data acquisition, data analysis, manuscript editing.

Nandita Melati Putri: definition of intellectual content, literature research, clinical studies, experimental studies, data acquisition, data analysis, manuscript editing.

Theresia Risa Davita: definition of intellectual content, literature research, clinical studies, experimental studies, data acquisition, data analysis, manuscript editing & review.

## Registration of research studies

1. Name of the registry: Research Registry
2. Unique Identifying number or registration ID: researchregistry5095
3. Hyperlink to the registration (must be publicly accessible): https://www.researchregistry.com/browse-the-registry#home/registrationdetails/5d6888c37a3579001058ba10/

## Guarantor

Hardisiwo Soedjana.

## Consent

Patient consent generaly applies to individual case reports rather than a cohort study.

## Funding

This research did not receive any specific grant from funding agencies in the public, commercial, or not-for-profit sectors.

## Declaration of competing interest

None.
